# Chance and purpose in the evolution of biospheres

**DOI:** 10.1098/rstb.2024.0085

**Published:** 2025-08-07

**Authors:** Philip C. J. Donoghue, Timothy M. Lenton, Samir Okasha, Graham A. Shields, Anja Spang

**Affiliations:** ^1^ Bristol Palaeobiology Group, School of Earth Sciences, University of Bristol, Bristol BS8 1TQ, UK; ^2^ Global Systems Institute, Faculty of Environment, Science and Economy, University of Exeter, Exeter EX4 4QE, UK; ^3^ Department of Philosophy, University of Bristol, Bristol BS6 6JL, UK; ^4^ Department of Earth Sciences, University College London, London WC1E 6BS, UK; ^5^ Department of Marine Microbiology and Biogeochemistry, NIOZ, Royal Netherlands Institute for Sea Research, AB Den Burg, The Netherlands

**Keywords:** evolution, biosphere, exo-biosphere, Gaia, biogeochemistry, selection

## 1. Introduction

The Earth system today is driven by an interlinked network of biogeochemical cycles that are governed largely by microbes [1]. This interplay between biology and geology both supports and is driven by the flourishing of life. But it was not always this way. Earth began as an abiotic geochemical system, and biogeochemistry has developed and complexified through geological time, driven in large part by the origin and assembly of novel microbial metabolisms.

Conventionally, the evolution of global biogeochemical cycles has been perceived as the fortuitous by-product of many happy accidents that have transformed a lifeless geochemical planet into what appears to be a self-regulating (homeostatic) biogeochemical planet [2]. The microbial metabolisms that drive global biogeochemical cycles have increased in diversity and complexity over time, creating recycling loops that prevent biolimiting elements from ending up in biologically unusable states [3]. An alternative view is that natural selection might promote the evolution of these cycles not just by favouring the evolution of new enzymes but also acting at the level of the cycle itself, favouring efficient and stable sets of interactions that persist through time. This hypothesis of biogeochemical evolution is founded in a growing body of work on multilevel selection, persistence-based selection and attempts to ‘Darwinize Gaia’ [4–6]. It provides a directly testable rationale for how the long-term complexification of the biosphere, the intertwining of metabolic networks, and the sense of purpose and directionality apparent from the history of life (documented by fossils and genomes) might have arisen from fundamental evolutionary principles. If this hypothesis is correct, the biogeochemical evolution of our planet has, perhaps predictably, been driven through directional selection towards resilient states of homeostasis, expanded upon through Earth history.

Reconciling competing views of chance versus directionality in the evolution of Earth and Life is challenging because we lack an integrated understanding of the biogeochemical evolution of our planet. This is not through lack of interest or effort. Indeed, the evolution of biogeochemical cycles and microbial metabolisms have been the focus of intense scientific investigation. However, the use of different data, methods and assumptions has often led to incompatible perspectives and conclusions. For example, some biogeochemical modellers consider all of the core metabolisms to have been in place before the last universal common ancestor (LUCA) [7], while biochemists infer LUCA as a ‘half alive’ acellular chimaera of geochemistry and biochemistry [8]. Others calibrate the evolution of metabolisms to patchy fossil and sedimentary geochemical evidence, indicating that these metabolisms evolved long after LUCA [9].

There has never been a better time to try and glean insight into the co-evolution of Earth and Life given the avalanche of genomic data [10] and key technical advances that enable its analysis. These include new probabilistic methods for mapping gene origins in evolutionary time, global biogeochemical models that can simulate the geochemical consequences of the sequential origin and spread of microbial genes and metabolisms, and autonomous evolutionary models that can explore the possible outcomes of life–planet co-evolution. Combining these approaches will provide us with a new understanding of how Earth evolved from a geochemical to a biogeochemical world, as well as a means to discriminate between the roles of chance and purpose in the evolution of Earth and Earth-like planets.

The aim of our meeting was to bring together these disparate perspectives and approaches to establish a common interpretative framework in which to research Earth’s evolving biosphere. These different perspectives exist principally because their proponents do not interact and instead effectively work within incompatible philosophies. The meeting facilitated that interaction, exposed inconsistencies between different perspectives and sought resolution between competing data and models of microbial, metabolic and biogeochemical evolution. This concerns both the historical pattern, written in palaeontological, geochemical and genomic records, but also the processes by which metabolisms, biogeochemical cycles and biospheres may evolve. The latter is of concern because the link between these hierarchical levels is unclear, as are the foci of selection that may or may not promote biospheric stability. This is problematic not only to evolutionary biologists, both empirical and theoretical, but also to philosophers of science who have long considered the veracity of multilevel selection theories. Most controversial of all is the idea that biospheric stability might be a state that biospheres inevitably evolve towards, which has implications for exobiology and the prospect of life elsewhere in the universe. By bringing together a diverse and intentionally critical group of experts, we hope to have made progress, not least in establishing a roadmap to an integrative understanding of the evolution of Earth’s biosphere and constraints on the modelling and identification of exo-biospheres.

## 2. Geological evidence of early life

Before considering hypotheses on the evolution of biospheres and their prevalence in the universe, it is pertinent to first consider the geological and biological evidence for evolution of Earth’s biosphere. Kasting & Ji [11] review current understanding of atmospheric oxygen and methane on the early Earth. These two biogenic gases are in an extraordinary state of thermodynamic disequilibrium in the atmosphere of the present day Earth [12], suggesting they may be actively regulated by life [13], but how have their concentrations varied over Earth history? Prior to life, atmospheric O_2_ levels would have been around one part in a trillion at the Earth’s surface. After the origin of oxygenic photosynthesis, O_2_ levels in the lower atmosphere remained below a part in a million, and methane levels could have been 2−4 orders of magnitude greater, thanks to the activity of methanogens. Thus, life maintained the late Archean atmosphere in a chemically reducing, disequilibrium state. Plumes of oxygen rising from ‘oxygen oases’ in the surface ocean may not have been well mixed throughout the atmosphere. In the Great Oxidation Event (or episode), 2.4−2.2 Ga, the atmosphere tipped irreversibly to an oxidizing state shielded by an ozone layer. Through the rest of the Proterozoic, Kasting & Ji [11] support the mechanistic view that O_2_ was regulated at around 10% of present atmospheric levels (or 2% of the atmosphere) by negative feedback from oxidative weathering [14]. Methane concentration would have been determined by its source flux from the recycling of primary production by methanogens and could have been hundreds of parts per million. Higher methane levels mean a stronger influence of methane on climate. Thus, when oxygen levels rose in the Great Oxidation and again at the end of the Proterozoic, transient drops in methane levels could have significantly cooled the climate and contributed to the low-latitude glaciations observed at these times. What does all this mean for biosphere evolution? It shows that at least since the origin of oxygenic photosynthesis, Life (the clade of all living things) has transformed the composition of the Earth’s atmosphere and exerted significant control on the climate. The resulting pattern of ‘punctuated equilibrium’—long intervals of one dynamic state of the system interspersed by shorter intervals of change to a different dynamic state—is in turn characteristic of a broader class of evolving self-regulating systems [15].

The fossil record provides our only direct insight into evolutionary history; however, interpreting the fossil evidence can be challenging, ranging from physical fossils, to sedimentary evidence of microbial activity, to geochemical evidence of metabolisms. Westall [16] reviews this diversity of evidence, describing possible evidence of iron-oxidizing bacteria in the Hadean followed by strong evidence of life, including anoxygenic photosynthesis from the Eoarchaean. There are records of diverse metabolisms by the Palaeoarchaean, including nitrogen fixation, sulphate reduction, methanogenesis, methanotrophy and phototrophy. There is convincing evidence of oxygenic photosynthesis in the Mesoarchaean, as well as iron cycling within the resulting oxygen oases, long before the shift from a dominantly anoxic to an oxic atmosphere in the early Palaeoproterozoic. Some of this evidence may constrain a younger origin of life on Earth than recent molecular timescales have estimated [17], but also an older origin of anoxygenic photosynthesis and oxygenic photosynthesis than others predict [18]. Nevertheless, Westall argues for a Hadean origin of life on Earth that is unrecorded by fossils principally because there is no rock record old enough from which they could be recovered.

The origin of eukaryotes is among the most formative events in evolutionary history. Some consider it so unlikely an event that it may represent a fundamental constraint on the emergence of complex life on other biospheres. There are many and varied hypotheses to explain the process of eukaryogenesis, often confused by competing definitions over what constitutes a eukaryote and the number of lineages that have contributed to their origin [19]. Almost every aspect of our understanding of the origin of eukaryotes is uncertain. Javaux [20] provides a broad perspective on this evolutionary episode, reviewing the fossil and biogeochemical record of eukaryogenesis, the sedimentary geochemical evidence of its environmental context and hypotheses on the timing of origin of crown eukaryotes, estimates of which range from the Neoarchaean to the Neoproterozoic. Javaux provides important new data that contributes to many of these issues, describing a diverse community of microfossils from the earliest Palaeoproterozoic of northern Australia. The assemblage includes a number of taxa that have previously been interpreted as eukaryotes, including *Dictyosphaera*, *Pterospersimorphita*, *Simia*, *Tappania* and *Valkyria*. However, these new materials are much older, some hundreds of millions of years older, than the most ancient records known hitherto. Javaux identifies characters suggestive of a crown-eukaryote affinity, including evidence of a complex cytoskeleton and endomembrane system. Based on association with fossil oxygenic cyanobacteria with preserved thylakoid membranes, Javaux argues some members of the assemblage must have possessed mitochondria. Together, these data and their interpretations point to an origin of crown eukaryotes before the Mesoproterozoic, within environments that are otherwise anoxic or suboxic.

## 3. Molecular evidence of early life and the evolution of metabolisms

Molecular phylogenies provide a means of seeing through the gaps in the rock and fossil records, and, hence, recent years have witnessed great progress in attempts to reconstruct the Tree of Life. The application of cultivation-independent sequencing-based approaches to characterize extant life on Earth has transformed our understanding of biodiversity. Particularly, the use of metagenomics and single-cell genomics has allowed researchers to reconstruct genomes of a vast and unprecedented diversity of uncultivated microorganisms and protists across the globe and provided a wealth of data to infer an updated tree of life based on gene sequences shared across the primary (i.e. Archaea and Bacteria) and secondary domain of Life (i.e. all eukaryotes including its single-celled members). The tree of life not only explains organismal relationships but also provides an important framework to establish a more systematic taxonomic classification system and reconstruct the evolution of lineages and their genomes through time from their LUCA. While the first universal phylogenetic tree established by Carl Woese [21] in 1987 only comprised 19 taxa, we now have access to thousands of genomes from across all domains of life. In this issue, Chen *et al*. [22] investigate how our understanding of the tree of life has changed during the past decades with an increasing number of taxa being added. Analysing such large datasets is computationally extremely challenging such that the authors implemented a recently developed software called uDance that relies on a divide-and-conquer algorithm to reconstruct phylogenetic trees with a large number of taxa [23]. A major observation of Chen *et al.* [22] is that while such large-scale approaches are valuable to visualize our current knowledge of biodiversity, they do not allow resolving critical evolutionary transitions such as the earliest transitions in the tree of life or the placement of eukaryotes. Indeed, accurately capturing evolutionary change through time and lineages requires the use of phylogenetic frameworks implementing complex evolutionary models that are currently not scalable to the vast amount of data available. Thus, the use of a taxonomically balanced set of high quality genomes from organisms across all major branches of the tree of life is more important than the addition of genomes from more shallow taxonomic levels for improving our understanding of the evolutionary history of life on Earth.

While downsampling taxonomic diversity has helped to improve species tree reconstructions and the earliest divergences in the tree of life, it limits resolution with regard to modelling the evolution of the diverse metabolic pathways and phenotypic diversity of microorganisms through time. Furthermore, these reconstructions require focusing on a very narrow set of marker genes that are shared across all Life and evolve predominantly vertically. However, horizontal gene transfer (HGT) is an important mechanism in microbial evolution with a recent estimate suggesting that more than 90% of bacterial gene families have been subject to horizontal transmission [24]. HGT contributes to the vast diversity of metabolic strategies characterizing members of the Archaea and Bacteria often even differing between closely related taxa. To better understand the interaction between the biosphere and geosphere, it is important to not only look at the evolution of clades but to reconstruct their metabolic repertoires and putative phenotypic features in light of biogeochemical constraints. In line with this, Padalko *et al.* [25] argue that universal trees fail to capture overall genomic diversity and thereby a comprehensive reconstruction of the evolution of metabolism. The challenge to predict phenotypic traits based on genomic predictions alone further limits our ability to connect organismal evolution to Earth’s history. The authors emphasize the importance of bioenergetics as a link between geochemistry and microbiology and point out several key aspects that need to be taken into account to improve our understanding of the evolution of energy metabolism: the mosaic nature of bioenergetic complexes and modularity and chimerism of enzymatic pathways as well as enzymes, and the dependency of the majority of bioenergetically relevant reactions on metals, prosthetic groups and coenzymes. They argue that to systematically elucidate bioenergetic evolution, phylogenetic approaches need to be combined with the experimental characterization of enzymes.

Thus, the search for the one true tree that accurately depicts life’s biodiversity as well as resolves the evolution of species and metabolic diversity remains challenging and an unfinished endeavour, with some of the most fundamental relationships still the focus of active research. Yet, several improvements in our understanding of deep cellular and metabolic evolution have been made in recent years. DPANN archaea and CPR bacteria were originally proposed to form sister groups of all other Archaea and Bacteria, respectively, and thus could have important implications for understanding cellular evolution and the LUCA. Members of the DPANN and CPR have reduced genome and cell sizes and incomplete biosynthetic pathways, living primarily as symbionts and so could be secondarily derived organisms. Mahendrarajah & Spang [26] attempt to resolve this issue by considering modern phylogenetic methods, which have revised the placement of the CPR bacteria as a derived sister group of Chloroflexota, indicating genome streamlining from an ancestor that was not obligately host-dependent. In contrast, while several different analyses have resolved the DPANN to be a sister group of all other Archaea, their monophyly and the exact root placement within the Archaea remains debated. They outline other new approaches, such as improved gene tree–species tree aware methods, determining gene family histories and integrating genome into evolutionary reconstructions of key metabolic pathways such as the Wood-Ljungdahl pathway, methanogenesis and anaerobic methane/alkane oxidation. Future advances will rely increasingly on multidisciplinary collaborations between biogeochemists, geologists, chemists, biologists and data scientists, integrating machine learning. Finally, resurrecting and experimentally studying ancestral proteins will allow us to better understand their function under simulated early Earth conditions and test predictions.

Despite the restrictions outlined above in reconstructing metabolic diversification, Moody *et al*. [27] tackle the problem of calibrating the evolutionary history of life to Earth’s geological record. For most of Earth’s history, life has been entirely microbial, and the major shifts in biospheric evolution were driven by the origin and evolution of microbial metabolisms. But microbes leave few interpretable fossil traces, so the main source of information on the evolution of their metabolism comes from comparative analyses of microbial genomes. Recent advances in phylogenetics deal with the problem of HGT and have been used to reconstruct rooted species trees for Archaea and Bacteria, thus permitting inferences about ancestral metabolisms to be drawn. Moody *et al*. [27] build on these advances to reconstruct ancestral gene repertoires for a subsampled but well-balanced set of prokaryotes. Their phylogenetic reconstructions enables them to draw inferences about the sequence in which modern microbial metabolisms were assembled, to deduce how the evolution of new metabolic genes has influenced the cycling of the core elements needed for life and to try to adjudicate between alternative models of biospheric evolution (such as entropic Gaia, Darwinized Gaia and a simple neutral model). Moody *et al*. find that the rate of metabolic evolution appears to have been higher earlier in the Earth’s history and that the rate of pathway assembly has varied between pathways cycling different key elements. An anaerobic carbon cycle was established early in life’s evolution, whereas biological cycling of elements such as nitrogen and sulfur began later, though nonetheless early in the Earth’s history. They confirm the finding that HGT has been a major force in microbial evolution, with Bacteria having higher rates than Archaea during the history of life; some pathways have been affected unevenly by HGT. Moody *et al*. [27] argue that a number of aspects of life’s evolution appear to be compatible with more than one model of biosphere evolution.

Another take on LUCA and the emergence of metabolism focuses on the evolution of protein catalysts that have likely always powered life by exploiting natural thermodynamic gradients. In their contribution, Nanda *et al.* [28] consider how metabolic reactions govern the flow of electrons and protons around the planet and are themselves dependent on the emergence and evolution of nanometre-scale chemical machines or proteins. In all extant life on Earth, oxidoreductases couple chemical energy from the environment with core redox reactions including photosynthesis, respiration and nitrogen fixation. The origins and emergence of complex life have been intimately tied with evolution of oxidoreductases. Nanda *et al.* [28] use structure-based analyses to describe the evolution of the protein catalysts in three biological epochs. First, thermodynamically driven polymerization reactions generated simple metal-binding peptides with specific sequences that catalysed core metabolic reactions. Second, these catalysts were incorporated into small structural ‘folds’ that served as building blocks for the third and final stage: extant, complex nanomachines. Although we have atomic-level structures for many core metabolic nanomachines, we do not yet understand how they originated, let alone how they work. This lack of understanding is especially obvious in at least two critical oxidoreductases: photosystem II, which is the machine that splits water from light, and nitrogenase, the machine responsible for generating ammonium from N_2_ gas. There are many additional nanomachines responsible for energy transduction and metabolism; however, the following two stand out: the origin and evolution of the ribosome, the machine responsible for making proteins, and the rotary ATPases, the machines responsible for generating cellular energy. All these core nanomachines are highly dynamic; that is, they functionally move at the molecular level to catalyse their respective reactions. While obviously there is much to be learned about the origin and evolution of life, fundamentally, we do not understand the rules for the emergence of functional dynamics in core proteins responsible for metabolism.

## 4. Oxygenation of the biosphere

Early metabolisms were limited by the energy that could potentially be released to power them, while nutrient limitation only became a dominant limitation much later. Stüeken [29] discusses where energy gradients were likely to have led to productivity hotspots before the introduction of oxygenic photosynthesis about 3 billion years ago, concluding that the early chemotrophic biosphere was dominated by volcanically active provinces fuelled by the injection of hydrogen and ferrous iron at hydrothermal vents. Once oxygenic photosynthesis arose, primary producers expanded first into freshwater habitats and, subsequently, into estuaries, in short where the flow of nutrients was both greater and more constant. In our search for life elsewhere in the galaxy, it may well be worth searching for signs of volcanism.

Horne *et al*. [30] consider the timing of origin of oxygenic photosynthesis and its impact, arriving at a surprising answer. While the Great Oxidation clearly depended upon the origin of oxygenic photosynthesis, they use a new biogeochemical model to show that the earlier oxygenic photosynthesis evolved, the longer the delay to a Great Oxidation. This counterintuitive result arises because availability of phosphorus—the ultimate limiting nutrient—controls the biogenic source of oxygen. Phosphorus ultimately derives from weathering of continental rocks and, in an ocean of oxygen oases, it is efficiently removed to organic sedimentary rocks that cannot oxidatively weather until after the Great Oxidation. This limits productivity and oxygen production both in the short term (within the ocean) and in the long term. The earlier that oxygenic photosynthesis starts, the more phosphorus is locked away in inaccessible form, while the later it starts, the more available phosphorus has already built up to fuel oxygen production. What does this counterintuitive result mean for Gaia? It shows that after the origin of oxygenic photosynthesis, self-regulation of a reducing atmosphere state was highly effective, due to a mix of biology and redox chemistry. But although productivity was boosted considerably by the origin of oxygenic photosynthesis, it was (inadvertently) self-limiting. From the perspective of obligately anaerobic prokaryotes this would have been a good thing, but from the perspective of aerobic life forms, including us humans, an early origin of oxygenic photosynthesis would have been very disappointing. We can be thankful for the late origin of oxygenic photosynthesis on Earth and indeed, from a weak Anthropic principle perspective, if this new model is correct, our existence requires that oxygenic photosynthesis evolved relatively late—as Horne *et al.* show early origin scenarios where a Great Oxidation is delayed indefinitely [30]. A late origin is *a priori* not so surprising given the inherent complexity of oxygenic photosynthesis involving capturing multiple photons to split water and two photosystems coupled in one cell [2]. On other Earth-like planets around more typical dimmer and redder M-type stars, the origin of oxygenic photosynthesis would be even more difficult [31]. Wherever it does eventually evolve, the new results predict an oxidized atmosphere will soon follow.

While recent work nevertheless indicates that oxygenic photosynthesis may already have evolved in the Archean or Proterozoic [24,32], currently available data suggest a late colonization of the open ocean by photosynthetic cyanobacteria around the Neoproterozoic–Phanerozoic boundary. In this issue, Braakman [33] reviews current frameworks on biospheric productivity in Proterozoic oceans and the metabolic evolution of planktonic marine cyanobacteria to better understand the apparent late evolutionary leap of these organisms from the benthos to the open ocean. Braakman points out that the availability of various nutrients such as nitrogen and phosphorus stimulating photosynthesis may have remained low in Proterozoic oceans low in oxygen, thereby limiting productivity. While arguments have been put forward to suggest that these constraints on productivity could have eventually loosened in increasingly oxygenated waters due to positive feedback loops and upon large-scale redox perturbations, Braakman favours the view that a ‘negative feedback built into the molecular machinery of photosynthesis’ may have prevented ocean oxygenation in the absence of biological inventions. In line with this, Braakman [33] reviews current evidence for the timing of cyanobacterial diversification and the evolution of their metabolic machinery that may have allowed these organisms to overcome earlier constraints on productivity. Furthermore, Braakman discusses the recently proposed ‘chitin raft hypothesis’ to explain the late colonization of cyanobacteria of the open ocean. In this scenario, the acquired ability of cyanobacteria to degrade chitin derived from the exoskeleton of diversifying Arthropods during the transition from the Neoproterozoic to the Palaeozoic is put forward as a key metabolic invention: specifically, Braakman [33] suggests that marine picocyanobacteria may have colonized chitin particles, which could have supported a surface-attached lifestyle and provided protection from stressors, eventually allowing colonization of the open ocean. Future research into this hypothesis and other unresolved questions regarding the expansion of cyanobacteria into ocean waters will be important to understand global patterns of biospheric productivity

## 5. Coevolution of Earth and Life

Through the Gaia hypothesis, Lovelock & Margulis [34] highlighted the homeostatic feedback loops that Earth exhibits, incorporating both Life and the lifeless components of the planet, presumably the consequence of evolution by natural selection. However, the view that homeostasis is more than a happy accident is controversial since this would imply that it has arisen as a consequence of higher-level selection. Critiques have bordered on parody, envisaging selection among populations of biospheres, some of which fail to make the cut [35]. Ultimately, however, the Gaia hypothesis is perceived to be unDarwinian because it fails to meet the codified expectations of evolution by natural selection, which emphasizes differential selection [36]. Doolittle [37] extends his attempt to Darwinize Gaia [4–6] by expanding the scope of selection to differential survival or persistence, not just of genes or organisms but to collectives including species, holobionts, ecosystems, lineages or clades. Doolittle reformulates Gaia as a lineage or a clade derived from LUCA and draws an analogy with clone survival in a chemostat, competing with other clones to be the ancestor of all future cells within the chemostat [38]. Within early Earth history, Doolittle envisages many wannabe LUCAs competing to persist. Our LUCA may have won out because of its shared biochemistry, which facilitated recycling loops for biolimiting resources and lateral gene transfer facilitating the persistence of linkages or steps within homeostatic processes. This is the basis of Doolittle’s ‘It’s the song not the singer’ (ITSNTS) theory [5], which underpins his Darwinization of Gaia.

Lenton [15] also defends a version of the Gaia hypothesis, though not in quite the same way as Doolittle. He argues that the Earth’s biosphere has increased its own ‘persistence and flourishing’ over time, in particular through greater free energy capture, more intense cycling of essential elements and the origin/enhancement of stabilizing mechanisms for atmospheric oxygen, temperature, ocean nutrients and carbon dioxide. Lenton describes the negative reaction of evolutionary biologists to the original Gaia hypothesis, which they saw as suspect given that the biosphere can hardly be supposed to have evolved adaptations for the good of the whole, akin to the adaptations of organisms, given the absence of selection at the level of biospheres. However, Lenton argues that there is no need to analogize the biosphere to an organism (as Lovelock originally did); rather, we should treat Gaia as a *sui generis* phenomenon. Even if we drop that problematic analogy, the stabilization of aspects of atmospheric composition, climate and ocean composition and the intense global cycling of essential elements are objective features that enhance the biosphere’s habitability and persistence and that are in need of explanation. Recent years have seen a resurgence of interest in trying to explain these feature in a way that is compatible with accepted scientific principles. Lenton identifies two different routes that such attempts have taken: a ‘physical pathway’ and a ‘biological pathway’ to Gaia. The former invokes general principles of complex systems theory and/or statistical physics. The basic idea is that any complex system is expected to evolve in the direction of increasing entropy; in the case of the biosphere, under some assumptions, this implies greater biomass, diversity and life-enhancing abiotic interactions. The biological pathway invokes what has been called ‘generalized selection’, a process that involves selection on entities that do not necessarily reproduce in the ordinary sense but nonetheless exhibit differential persistence. These entities may be ecosystems, nutrient cycles or clades, for example. The idea is that selection on these sub-biospheric-level entities, as a by-product, may lead the biosphere as a whole to exhibit Gaia-like properties. Lenton argues that the physical and biological pathways to Gaia are converging and may mutually reinforce each other: both entropy and selection play a role in explaining key biospheric properties.

Boyle [39] also examines the status of the Gaia hypothesis, but from a different perspective. He argues that the Gaia hypothesis should be distinguished from the general idea that Earth and life are interconnected and mutually affect each other, which can hardly be disputed. As Boyle [39] understands it, the Gaia hypothesis proposes that the Earth has evolved ‘planetary scale’ attributes that promote its habitability and that those attributes are causally connected to the presence of life itself. The standard neo-Darwinian objection to this hypothesis, of course, is that there is no way that such features could have evolved by natural selection, since there is no reason to think that habitability-promoting genotypes would enjoy a higher long-run growth rate that habitability-inhibiting genotypes; thus, any planetary attributes that happen to promote habitability are simply fortuitous by-products. Boyle [39] examines the variety of attempts that have been made to evaluate Gaia by critics and proponents alike. He divides these into three camps: ‘reject’, ‘accept without Darwinizing’ and ‘Darwinize’. Those in the ‘reject’ camp claim that the Gaia hypothesis is simply false and leave it at that. However, this leaves unexplained the empirical observations that motivated Gaia in the first place, such as the continuous occurrence throughout Earth’s history of (broadly) life-permitting temperatures, allowing the ongoing presence of liquid water, and the biologically driven recycling of biologically essential elements. Those in the ‘accept without Darwinizing’ camp hold that the Gaia hypothesis is correct; however, they try to explain the existence of habitability-promoting attributes without appeal to natural selection, for example, by arguing that complex systems have an inherent tendency to produce stability and that Gaia is just a special case of this, or by invoking so-called ‘sequential selection’. Boyle finds these attempts to offer non-Darwinian explanations of Gaia unsatisfactory. Those in the ‘Darwinize’ camp try to show that the Gaia hypothesis can in fact by reconciled with a form of natural selection, despite the early neo-Darwinian critique. Typically, this involves appealing to selection above the level of the organism, e.g. at the clade level, and/or arguing that ‘persistence selection’ can operate on entities that do not reproduce their kind. Doolittle’s ITSNTS theory [4–6] is a recent example of a theory in this third camp. Boyle [39] offers a sympathetic exposition of the ITSNTS theory but suggests that exoplanet exploration will ultimately be needed to determine the veracity of the Gaia hypothesis and its compatibility with Darwinism.

Hermida & Okasha [40] question whether there is evidence for planetary homeostasis at all, arguing that it is not required to explain the long-term habitability of Earth. In their view, active planets should be expected to achieve a stable steady state of energy flow through their systems, and this does not require life or homeostatic effects provided by life. Rather, it is the persistence of habitability that is the real phenomenon to be explained, and they set out to determine whether this is the consequence of selection (or a selection-like phenomenon) at the level of the biosphere or whether it is a by-product of selection at lower levels. In considering attempts to Darwinize Gaia, Hermida & Okasha [40] reflect on previous attempts to invoke higher-level selection, insisting on a distinction between true group adaptations and group-level by-products arising from selection on individual organisms. Ultimately, they conclude that, in the absence of a population of, say, biogeochemical cycle variants, there can be no higher-level selection and so no higher-level adaptations. Indeed, there is certainly no population at the level of the biosphere, which is unique. It is possible that the clade of extant life was once part of a broader population within which there was competition or, at least, selection, but they existed early in Earth history, before the emergence of the biospheric properties that Gaia theory seeks to explain. Furthermore, higher-level selection cannot explain the origin of those clades. Hermida & Okasha [40] also consider Lenton *et al.*’s proposal of sequential selection as a process underpinning the long-term maintenance of planetary habitability [41]. In this formulation, life has positive and negative effects upon life-supporting characteristics of a biosphere, leading either to a steady state or else resets or extinction when the bounds on planetary habitability are plumbed. However, Hermida & Okasha [40] consider this a Lamarckian transformational mechanism that does not draw upon variation and explains only the nature of a single individual, rather than a member of a population. Ultimately, they conclude that Darwinized Gaia requires that higher-level traits are adaptations, not by-products, and subject to evolution by natural selection at that higher level. They find no such evidence, and they remain unpersuaded by these attempts to Darwinize Gaia.

## 6. The evolution of biospheres and the search for exo-biospheres

Seager *et al.* [42] pick up the question of the diversity of exoplanetary atmospheres and possible biospheres. Remarkably, as Seager showed at the discussion meeting, it is now possible to observe a transiting exoplanet atmosphere, albeit most readily that of a large gaseous planet around a small faint star. Still, the limits of current technology are being pushed to try and resolve the atmospheres of Earth-like planets around small red dwarf stars (e.g. Trappist-1). What atmospheres could exist and support life? Seager *et al.* [42] suggest numerous options. H_2_-dominated atmospheres are favourable for detection, although to retain them requires a more massive planet than Earth, and autotrophic life can live on H_2_ and a source of carbon. Carbon monoxide (CO) could accumulate on rocky exoplanets, and prokaryotic life can live off CO as a source of both energy and carbon. While CO is toxic to mammals, it is not toxic to all animals and thus does not preclude complex life. CO_2_-dominated atmospheres can support life as one did on the early Earth. O_2_-dominated atmospheres may be produced abiotically by photodissociation and loss of hydrogen during a runaway greenhouse phase, and several prokaryotes can survive in a pure O_2_ atmosphere. However, it would pose toxicity and flammability challenges, and a runaway greenhouse means extreme heat and desiccation. Seager *et al.* [42] go further and suggest that solvents other than water could support life and that a biosphere might conceivably originate in clouds. However, contact with a rocky surface, or an abundant supply of meteorites, is usually seen as necessary to provide access to metal ions. Clearly there are more atmospheric options for an exo-biosphere than a ‘terracentric’ perspective would suggest. But would such biospheres be distinguishable from abiotic null cases? This brings us back to Lovelock’s [12] generic approach of looking for extreme atmospheric disequilibrium as the signature of Life, but to create detectable disequilibrium in turn requires high productivity. This invites modelling research on the potential productivity and effects on atmospheric composition of the exo-biospheres proposed by Seager *et al*. [42].

Lineweaver & Chopra [43] provide an interesting counterpoint to Seager *et al.* [42] by emphasizing the potential for young exo-biospheres to be eliminated in a ‘Gaian bottleneck’ of runaway greenhouse or runaway ice-albedo effects. This familiar ‘terracentric’ perspective assumes liquid water is a necessary condition for life and does not consider options such as an O_2_-rich atmosphere created from a runaway greenhouse being habitable. Rather than there being many options for Life, Lineweaver & Chopra argue that habitable worlds tend towards an uninhabitable state, and if Life does get started, it must rapidly establish Gaian regulation of its host atmosphere and climate or be extinguished. They consider several specific mechanisms by which early Life could act to influence or stabilise climate, including by altering methane levels (as noted by Kasting & Ji [11]) or by getting involved in damping feedbacks involving silicate weathering or low cloud cover. But this does not answer the more fundamental question of why the net result would tend to be stabilizing (rather than destabilizing). Here Lineweaver & Chopra join Doolittle [4,37] in arguing that the clade of Life can acquire the adaptations needed to get through a ‘Gaian bottleneck’ through persistence-based selection. Thus they conclude Darwinized Gaias are rare but plausible. Existing, generic exo-biosphere simulations show that indeed biospheres can suffer Gaian bottlenecks and some can get through them by tending to regulate atmospheric composition and climate [44]. However, the same modelling shows that several other general paths of biosphere evolution are plausible that lack a bottleneck [44].

The question of whether life is present on other planets is a perennial source of intellectual fascination and the central topic of exobiology. So too is the question of whether intelligent, complex life (as opposed to ‘mere’ microbial life, for example) is present on other planets. Watson [45] starts with the observation that given the present state of science, we know for sure only that life, and hence intelligent life, has arisen on one planet, namely Earth. However this does not prevent us from getting a handle on the above questions, by considering how easy or difficult it was for intelligent life to evolve on Earth. To address this, Watson revisits the ‘hard steps model’ for the evolution of complexity first introduced by Carter [46], whose key assumption is that complexity requires the occurrence of a few intrinsically unlikely events, or hard steps, that occur at random times. All other steps are easy, so are assumed to occur sufficiently fast that they have no impact on the long-term rate of increase of complexity. The model allows us to derive probability distributions for the timing of each hard step, conditional on complexity actually having evolved. The hard steps model can apply to any sequence of unlikely events; one interesting application is to the evolution of humans and, by extension, intelligent life on other planets. Watson considers a number of candidates for what the hard steps for this case may have been, arguing that the origin of oxygenic photosynthesis and the origin of eukaryotes are the two events that best fit the bill. He then modifies the hard steps model by introducing ‘delays’ between the occurrence of a hard step and its downstream consequences that facilitated the next step. The modified hard steps model make a number of predictions, in particular the prediction that, even if biogenesis is relatively easy and microbial life of some sort quite common in the universe, we are unlikely to ever find Proterozoic or Phanerozoic-like biospheres, in virtue of the fact that oxygenic photosynthesis is a hard step.

Nicholson [47] takes two model biospheres in order to inform the search for alien life. The first model is of a simple methanogen biosphere and demonstrates that the concentration of a biogenic gas in the atmosphere is only very minimally impacted by population dynamics, which reduces the number of assumptions needed to predict biosignatures for a given metabolic pathway. The second model is of an evolving life–environment coupled system, or a Tangled Nature Model, which demonstrates that the most probable trajectory for an exo-biosphere is to diversify over time due to a mechanism known as Sequential Selection with Memory. Nicholson makes the case that while bio-signature searches are still costly and time consuming, modelling has an important role to play in establishing useful principles that can be used to guide searches and limit false positives.

## 7. Implications of and for human activities

Since the 1950s, our planet entered a new phase of conscious stewardship. Industrialization not only polluted but awakened a shared environmental conscience that rejects pollution as an inevitable consequence of human ambition. It is easy to forget that toxic pesticides, heavy metals and radioactive nuclides, to name but a few, were all targets of global mitigation efforts. Although the effects of carbon dioxide are more insidious, Vince [48] presents climate mitigation as another environmental issue in need of global collaboration. This adds a human dimension to the question of purpose in the evolution of biospheres, which she explores through her intriguing concept of the ‘extended biosphere’, extended in the same way that our minds and bodies have become extended through technological advances. We humans undoubtedly modify the world we live in, but intentional intervention on a planetary scale is a relatively recent phenomenon. While negative impacts, such as pollution, global warming or acidification, have been unintentional consequences of industrialization, rebalancing has been wholly intentional. Indeed, environmental stewardship has gone hand-in-hand with the ‘Great Acceleration’ in population, living standards and industrialization that began in the 1950s and is based on a deeper understanding of the interconnectedness of life and the physical environment. Vince’s opinion piece represents a call to action not only for systemic intervention on a global scale but also a plea for debate, objectivity and shared responsibility.

## Data Availability

This article has no additional data.
